# Reference gene selection for quantitative real-time PCR (qRT-PCR) expression analysis in *Galium aparine* L.

**DOI:** 10.1371/journal.pone.0226668

**Published:** 2020-02-04

**Authors:** Xu Su, Liuyang Lu, Yashe Li, Congai Zhen, Guilei Hu, Kun Jiang, Yawei Yan, Yanbo Xu, Geng Wang, Mingwang Shi, Xiling Chen, Baizhong Zhang

**Affiliations:** 1 College of Resources and Environment, Henan Institute of Science and Technology, Xinxiang, P.R. China; 2 The Museum of Chinese Gardens and Landscape Architecture, Beijing, P.R. China; Ankara University, TURKEY

## Abstract

To accurately evaluate expression levels of target genes, stable internal reference genes is required for normalization of quantitative real-time PCR (qRT-PCR) data. However, there have been no systematical investigation on the stability of reference genes used in the bedstraw weed, *Galium aparine L*. (BGA). In this study, the expression profiles of seven traditionally used reference genes, namely *18S*, *28S*, *ACT*, *GAPDH*, *EF1α*, *RPL7* and *TBP* in BGA were assessed under both biotic (developmental time and tissue), and abiotic (temperature, regions and herbicide) conditions. Four analytical algorithms (geNorm, Normfinder, BestKeeper and the ΔCt method) were used to analyze the suitability of these genes as internal reference genes. RefFinder, a comprehensive analytical software, was used to rank the overall stability of the candidate genes. The optimal normalization internal control genes were ranked as: *28S* and *RPL7* were best for all the different experimental conditions (developmental stages, tissues, temperature, regions and herbicide treatment); *28S* and *RPL7* for developmental stages; *TBP* and *GAPDH* for different tissues; *28S* and *GAPDH* were relatively stable for different temperature; *28S* and *TBP* were suitable for herbicide treatment. A specific set of reference genes were recommended for each experimental condition in BGA.

## Introduction

Quantitative Real-time PCR (qRT-PCR) is considered as the most common method for gene expression quantification, due to high sensitivity and efficiency[1–3]. However, the accuracy of qRT-PCR can be influenced by the quality of the template, the efficiency of transcription and amplifcation, and experimental procedures between samples[4]. To enhance the accuracy of qRT-PCR analysis, several strategies such as normalization of sample size, ensurence of the RNA quality and quantity, and removal of DNA contamination were carried out [5]. Among these strategies, normalization of gene expression is essential to prevent nonbiological alteration or incorrection, and the currently preferred practice is to determine the target gene expression with an internal reference gene as control[1, 6]. No reference gene exhibits stable expression under all conditions. Therefore, it is essential to select stable reference genes for determining the transcript changes of target gene by qRT-PCR. Besides, it is integral to estimate suitable reference genes for each specific condition. Several algorithms, such as geNorm[7], BestKeeper[8], and NormFinder[9]were used for candidate reference genes evaluation. The frequently used housekeeping genes, such as β-actin (*ACT*), α-tubulin (*TUA*), ubiquitin (*UBQ*), cyclophilin (*CYP*), glyceraldehde-3-phosphate dehydrogense (*GAPDH*), *18S* or *28S* ribosomal RNA and elongation factors (*EF*) have been verified to be suitable as internal control genes in many plants[10–12].

Bedstraw weed, *Galium aparine L*. (BGA), a malignant weed from the Rubiaceae family, can cause great harm to oilseed rape, wheat and some other crops in China[13]. The control of this weed normally relies on chemical herbicides for decades. However, the wide, unreasonable and dominant application of chemical herbicides not only caused cropland deterioration, but also caused rapid development of weed resistance to chemical herbicides[14]. Resistance mechanisms usually included target-site resistance (TSR) and/or non-target site resistance (NTSR) mechanisms. Both the over-expression of target proteins and structural changes to the herbicide-binding sites belonged to TSR mechanisms. Hence, it was necessary to select the suitable reference genes for evaluation the target gene expression in weed species. To date, the expression levels of reference genes have been commonly quantified in various weeds by qRT-PCR[15,16]. Only one reference gene such as *18S*, *GAPDH*, or *ACT* was selected for normalization the target gene expression by qRT-PCR under all the diverse experimental conditions in multiple studies. However, it is indispensable to use at least 2 or 3 reference genes for achieving accurate normalization[7,17].

Reference gene validation in weeds is lacking of systematization and should be paid more attention, especially in agricultural weeds. Hence, it is important to assess stable reference genes in BGA for qRT-PCR quantification. Seven common reference genes, such as *18S*, *28S*, *ACT*, *GAPDH*, *EF1α*, *RPL7* and *TBP*, were selected and evaluated for target gene expression normalization using the qRT-PCR method in BGA. Our results revealed that specific reference genes were required for qRT-PCR normalization under various experimental conditions.

## Materials and methods

### Cultivation of seedlings

The seeds of BGA used in this study were collected from a Xinxiang county wheat field (35°18'13.71''N, E113°55'15.05''E) in Henan Province, China, in Year 2010. The wheat field belonged to our college and collection was permitted by our college. Cultivation of BGA was adopted by the greenhouse potting methods[18]. The seeds of BGA were sown into 75 cm^2^ pots filled with a mixture of soil sand and grass biochar, and germinated in the greenhouse under 20 / 15 °C day/night temperatures with a 12 /12 h (light /dark) cycle and 75±5% relative humidity.

#### Biotic conditions

BGA foliar parts were obtained with specific developmental stage including 1-leaf stage, 2-leaves stage, and 3-leaves stage. Different vegetative tissue including leaves, roots, and stems were seperately collected. Ten individual plants was randomly selected per repetition and three biological replicates were used in this study. After collection, all samples were rapidly frozen in liquid nitrogen and stored at -80 °C for subsequent total RNA isolation and qRT-PCR testing.

### Abiotic conditions

To examine the effect of herbicide, the concentration of herbicide tribenuron-methly was determined as 10 g ai/ha (IC_10_) by a modified whole-plant assay[19]. 3-leaves stage BGA plants with height about 20 cm were exposed to tribenuron-methly at the corresponding IC_10_ concentration with an auto spray device (ASP-1098) under 0.2 MPa pressure. The liquid volume was 450 L each ha. Water alone was selected as control. Foliar parts were collected after 24 h treatment, quickly frozen with liquid nitrogen before storage at −80 °C.

### Selection of reference gene and primer design

To identify suitable reference genes for qRT-PCR analysis in BGA, seven common reference genes, such as 18S ribosomal RNA (*18S* rRNA), 28S rRNA (*28S*), actin (*ACT*), elongation factor 1 alpha (*EF1α*), glyceraldehyde-3-phosphate dehydrogenase (*GAPDH*), ribosomal protein L7 (*RPL7*), and TATA box binding protein-associated factor (*TBP*), were selected for qRT-PCR analysis. The primers used for qRT-PCR were designed with the Primer3.0 software. The corresponding primer information were listed in Table 1. Besides, the detailed sequences of the seven selected reference genes were shown in S1 File.

**Table 1:** Primer information of candidate reference genes and a target gene.

Gene Symbol	Gene name	Tm (°C)	Sequence (5´–3´)	Product Length (bp)
*ACT*	Actin	57.45	F: GGACCGGTTGAGTTCGAGAA	111
		55.40	R: ACTACGGCCAACCTTGTCAA	
*18S*	18s ribosomal	57.45	F: GCAGTCTCCATCAACCCACT	106
		57.45	R: CTTCACCGCTAACATCACGC	
*28S*	28s ribosomal	55.40	F: TTGTCCGCATCAAAACTGGG	98
		55.40	R: AACGACTATTCCGGCACTCT	
*GAPDH*	Glyceraldehyde-3-phosphate	55.40	F: GCCCATTTTCAGCTGCAAAC	117
		55.40	R:TTTGCAGCCAACTTCTTCCC	
*EF1α*	Elongation factor 1 alpha	57.45	F:TCTGACCGTCCTTGGAGATG	93
		57.45	R: TGATCACCGGAACCTCTCAG	
*TBP*	TATA box binding protein	57.45	F: TTGGGTGTTACTGTGGAGGC	112
		57.45	R: CAGTGGCTGGAATGGAAGGA	
*RPL7*	Ribosomal protein L7	55.40	F: AAGACGAAGGAGCTGCAGAA	106
		55.40	R:CAACTTCTATGTGCCCGCAG	
*HSP70*	Heat shock protein 70	58.98	F: ACCTGCACCTGATGTCGTTA	105
		58.93	R: GGTGCTGCTTCCTTCTTCTG	

Notes: F, forward primer; R, reverse primer; Tm, melting temperature; R^2^, coefficient of determination

### Total RNA extraction and cDNA synthesis

Total RNA was isolated using TRIzol (Invitrogen, USA) according to the manufacturer’s recommendations. The RNA integrity was determined by electrophoresis with 1% agarose gel. The quality and quantity of RNA were measured using a NanoDrop 2000 UV-Vis spectrophotometer (Thermo Fisher Scientific Inc., USA). Each sample of 1 μg RNA was reverse transcribed using PrimeScript^™^ RT reagent Kit with gDNA Eraser (Perfect Real Time) (TaKaRa, Japan) following the manufacturer’s instructions in a total volume of 20 μl. The cDNA templates were stored at -20°C until use in qRT-PCR.

### Quantitative real-time PCR (qRT-PCR)

qRT-PCR was implemented in a 7500 Real-Time PCR system (Applied Biosystems, USA). Each reaction (20 μl) contained 1 μl of cDNA template, 10 μl of SYBR Green qRT-PCR SuperMix-UDG (Invitrogen, USA), 0.3 μl of each specifc primer, and 8.7 μl of nuclease-free water. No cDNA template but corresponding volume nuclease-free water was used as NTC.

The amplification conditions for qRT-PCR were set as follows: 50°C for 2 min, 95°C for 2 min, and 40 cycles of 95°C for 15 s, and 55°C for 30 s. For melting curve analysis, a dissociation step cycle (from 65 to 95 °C) was carried out to verify the gene-specific amplifcation. Relative standard curves for the transcripts were conducted with a 5-fold dilution series of cDNA. The corresponding qRT-PCR efficiencies (*E*) were calculated using the equation: E = (10^−1/slope^-1)×100% [20–22]. All opperations were processed with three biological replications and at least two technical replications.

### Constancy analysis of reference gene expression

The stability of the seven candidate reference genes was assessed using the comparative ΔCt method[23], geNorm[7], NormFinder[9], and BestKeeper[8]. RefFinder, one free available online software was also applied to further estimate the stability of reference genes with the advantage of integrating the above all four major computational programs.

The geNorm program provides a calculation of gene expression stability value (M), and the reference gene with the lowest M value is considered as the most stable gene. In general, the candidate reference gene is considered stable when an M value below 1.5. The geNorm also performs a pairwise variation (V) with a serial ratio of V_n/n+1_. Addition of another reference gene could be required for accurate normalization when a V_n/n+1_ value above 0.15. NormFinder software calculates the expression stability of the candidate reference gene in all given sample set and ranks the stability order of candidate genes[9]. BestKeeper considers the Ct values of all the selected reference genes to determine the standard deviation for screening the “optimal” reference genes. The ΔCT method directly make a relative expression comparison between two candidate genes within the same sample. RefFinder combines the above four methods and gives the overall final ranking order.

### Validation of selected reference gene

One heat shock gene (*HSP70*) was regarded as the target gene to validate the reference genes stability. *HSP70* expression levels under diverse factors were assessed according to different candidate reference genes as the internal control genes. Normalization factors (NFs) were computed based on the geometric mean values which was determined by RefFinder. The expression profiles of *HSP70* in different treatment were conducted with the 2^–ΔΔCT^ method[20].

### Statistical analysis

Statistical analysis were performed with the software InStat v.3.0 (GraphPad Software, San Diego, CA). One-way ANOVA test was chosen for different comparison of target gene expression with significance *P* < 0.05.

## Results and discussion

### Evaluation of reference genes expression stability

All the primer pairs of seven selected candidate reference genes and one target gene (*HSP70*) was specific through evaluation by PCR. For each candidate reference gene, a single PCR product band with the expected size was visualized by 1.5% agarose gel. Besides, gene-unique amplification was also corroborated by a single qRT-PCR melting curve peak. Standard curve analysis revealed that the amplification efficiency of qRT-PCR varied from 90.5 to 105.3% (Table 1). Besides, the linear regression correlation coefcients (R^2^) of all the 8 genes ranged from 0.983 to 0.998 (Table 1).

**Fig 1:**
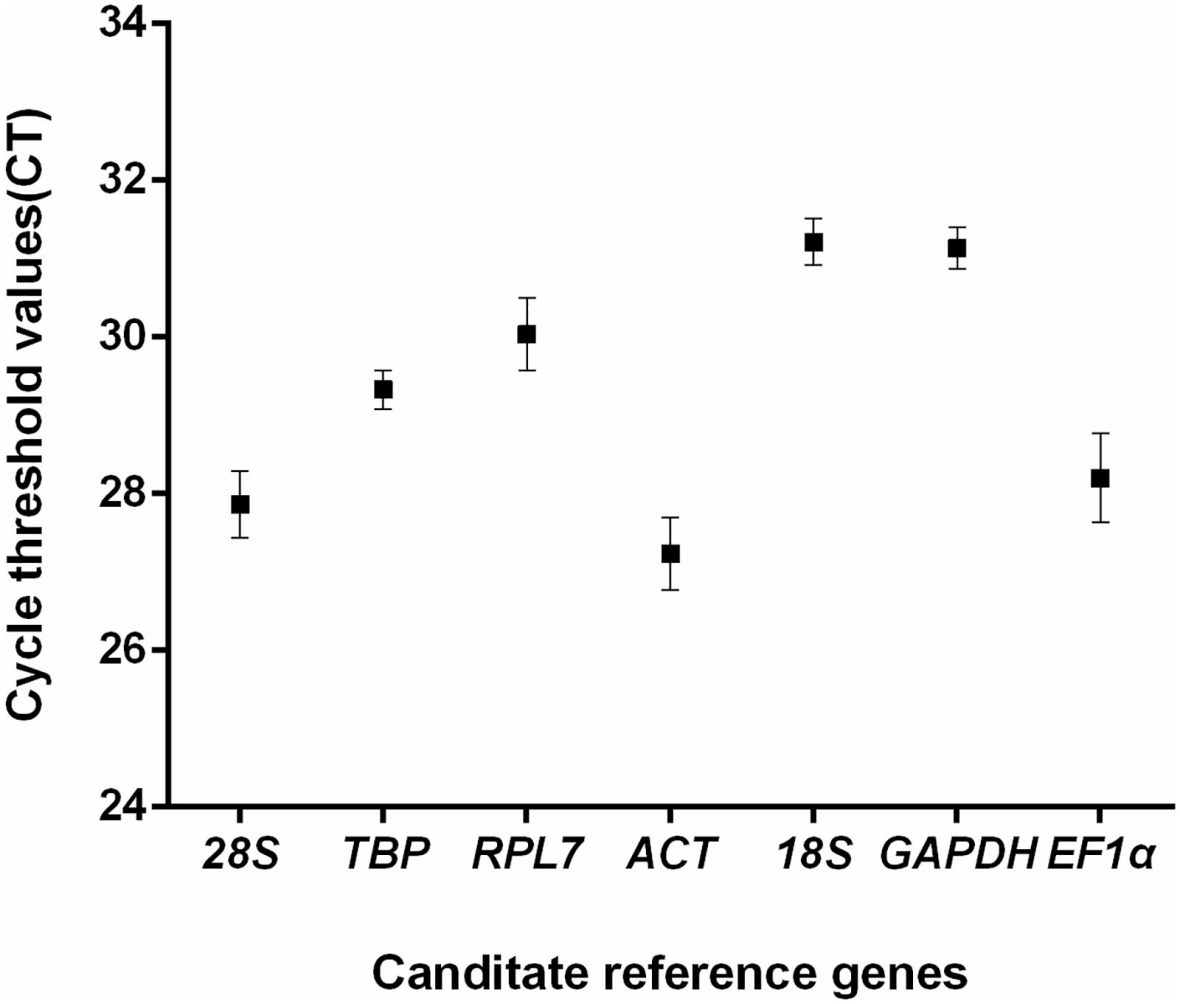
Expression levels of candidate reference genes of *Galium aparine*. The mean Ct values of candidate reference genes in all tested samples were indicated by the black dot, while the standard deviation of the mean was represented by the bars.

The mean Ct values of all the tested reference genes were between 27.24 (*ACT*) to 31.21(*18S*), covering all the experimental conditions (Fig 1). Among the candidate genes, *ACT* was the most accumulated transcripts, indicated by the lowest mean Ct value of 27.24, whereas *18S* had the lowest transcript expression level with a mean Ct value of 31.21.

**Fig 2:**
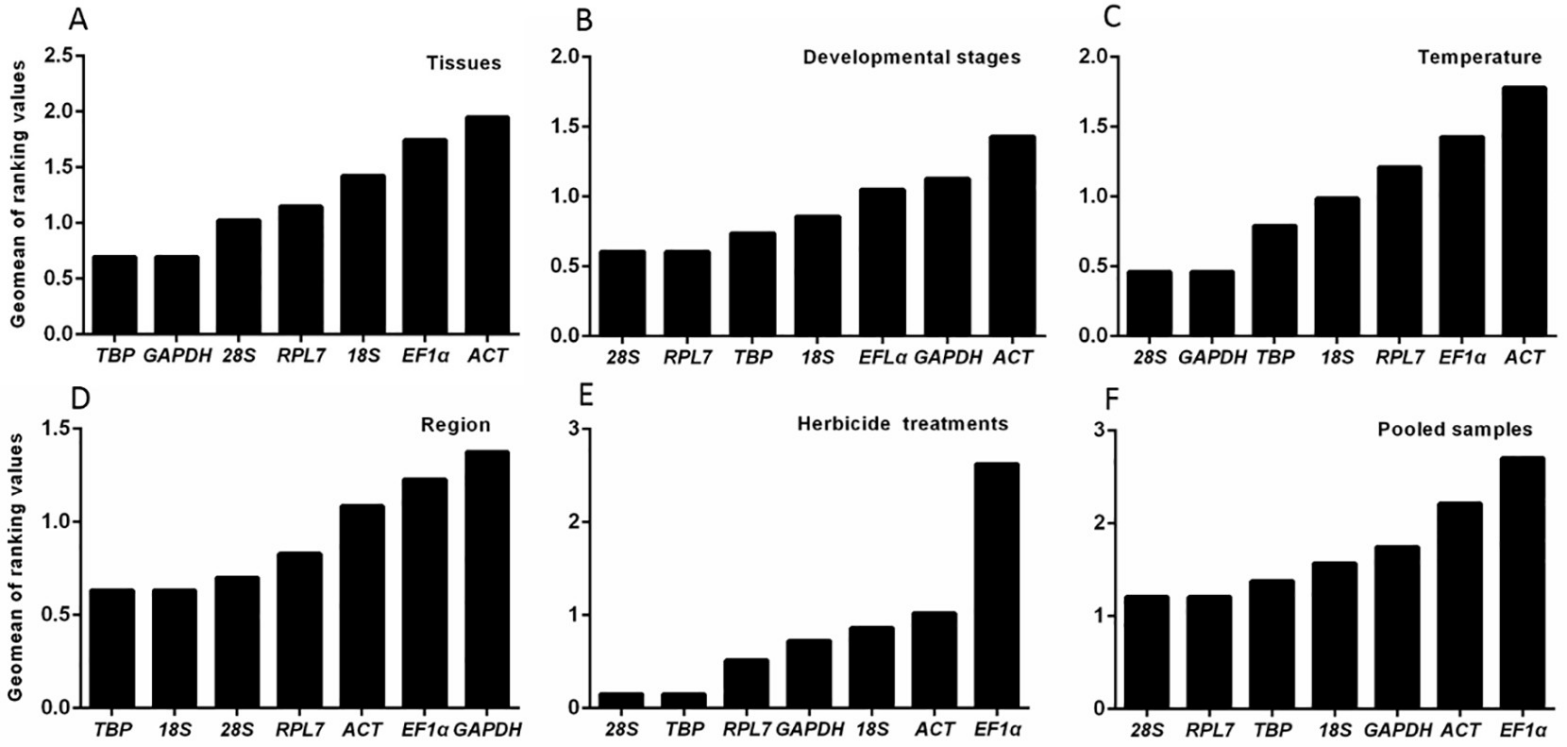
Expression stability of the candidate reference genes in response to different conditions. The average expression stability of the reference genes as calculated using geNorm. A lower Geomean of ranking value indicates more stable expression. (A) Tissues, (B) Development stage, (C) Temperature, (D) Regions, (E) Herbicide treatments, (F) Pooled samples.

**Fig 3:**
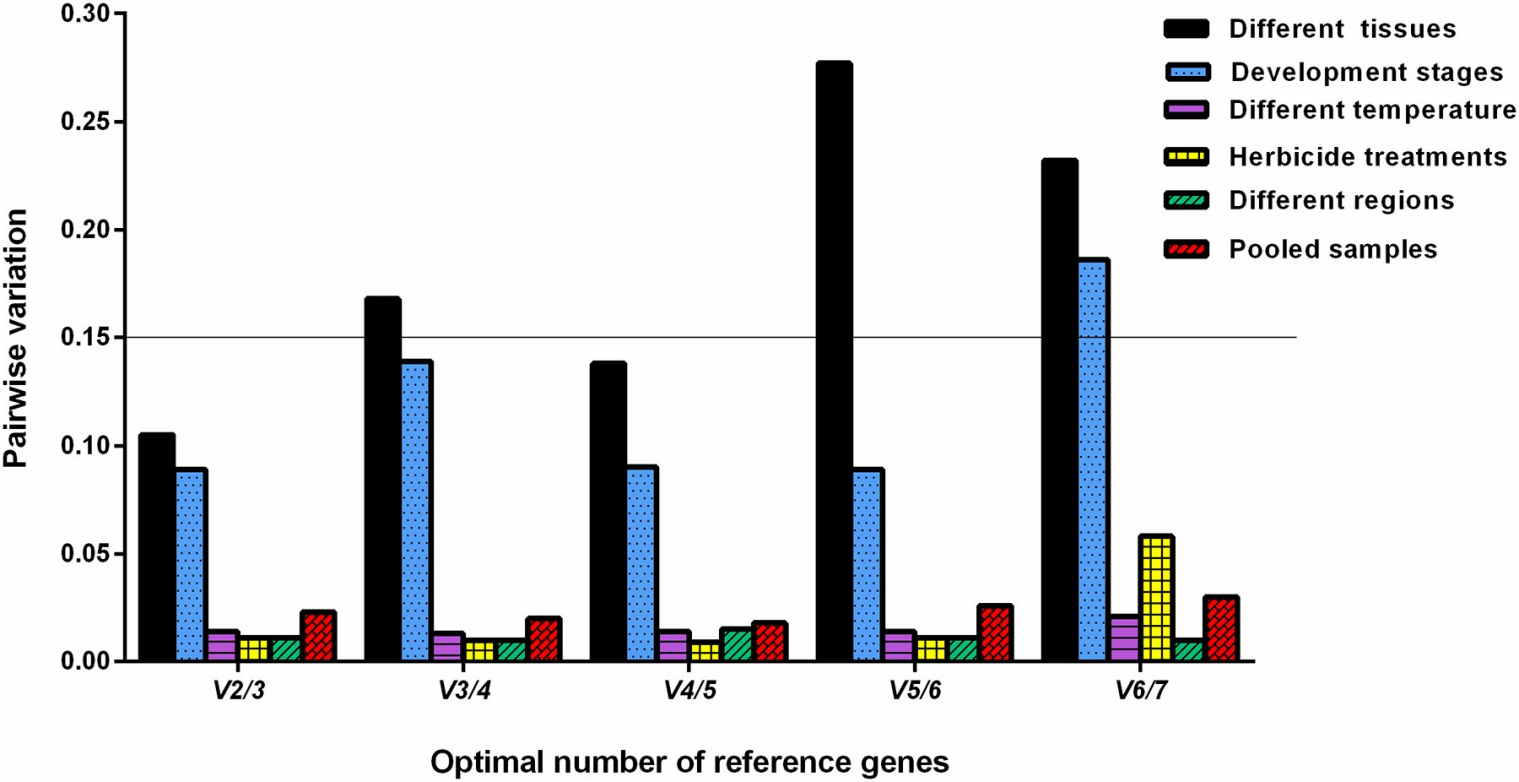
Optimal number of reference genes for normalization in *Galium aparine*. The normalization factors (NF_n_ and NF_n+1_) was employed to analyze pair-wise variation (V_n/n+1_), then it can determine the optimal number of reference genes required for accurate normalization in a given set of experiment. A value < 0.15 indicates that the use of additional reference genes would not markedly improve normalization.

For tissue samples, the rankings of reference gene stability were similar based on ΔCt method and Normfinder, showing *TBP* and *RPL7* as the top two most stable genes. While, *TBP* was considered as the most stable gene using BestKeeper and geNorm (Table 2). RefFinder produced the stability rankings from most stable to least as *TBP*, *GAPDH*, *28S*, *RPL7*, *18S*, *EF1α* and *ACT* (Fig 2A). GeNorm indicated that the gene number would be suitable for normalization. In comparison with the cut-off value of 0.15, the V_3_/_4_, V_5_/_6_, and V_6_/_7_ values were exceeded (Fig 3), indicating that additional reference genes are required.

**Table 2:** Expression stability of the candidate reference genes in response to different conditions.

Conditions		ΔCt		BestKeeper		Normfinder		geNorm	
		Stability	Rank	Stability	Rank	Stability	Rank	Stability	Rank
Different tissues	*ACT*	2.455	7	2.462	7	2.138	7	1.946	7
	*18S*	2.185	5	1.288	5	1.776	5	1.426	5
	*28S*	1.942	4	0.636	3	1.469	4	1.023	3
	*GAPDH*	1.705	3	0.565	2	1.081	3	0.696	1
	*EF1α*	2.253	6	2.068	6	1.840	6	1.745	6
	*TBP*	1.525	1	0.403	1	0.644	2	0.696	1
	*RPL7*	1.570	2	1.181	4	0.352	1	1.148	3
Developmental sages	*ACT*	2.183	7	1.402	7	2.085	7	1.429	7
	*18S*	1.420	5	1.170	6	1.058	5	0.858	3
	*28S*	1.250	3	0.758	4	0.763	4	0.604	1
	*GAPDH*	1.325	4	0.397	1	0.759	3	1.128	6
	*EF1α*	1.215	2	0.538	2	0.435	1	1.049	5
	*TBP*	1.173	1	0.725	3	0.570	2	0.738	2
	*RPL7*	1.438	6	1.091	5	1.185	6	0.604	1
Herbicide treatments	*ACT*	2.234	6	1.235	4	1.224	5	1.022	6
	*18S*	2.226	5	1.641	6	1.656	6	0.864	5
	*28S*	1.701	2	0.863	2	0.075	1	0.150	1
	*GAPDH*	1.990	4	0.610	1	0.376	3	0.723	3
	*EF1α*	6.638	7	4.652	7	6.605	7	2.626	7
	*TBP*	1.691	1	0.867	3	0.075	2	0.150	1
	*RPL7*	1.904	3	1.383	5	1.055	4	0.513	2
Temperature	*ACT*	2.660	7	2.284	7	2.476	7	1.779	7
	*18S*	1.406	1	0.906	2	0.529	1	0.986	3
	*28S*	1.432	2	1.480	5	0.566	2	0.460	1
	*GAPDH*	1.560	4	1.529	6	0.978	4	0.460	1
	*EF1α*	2.041	6	0.701	1	1.657	6	1.427	6
	*TBP*	1.522	3	1.299	4	0.886	3	0.791	2
	*RPL7*	1.836	5	0.931	3	1.453	5	1.210	5
Region	*ACT*	1.476	5	1.410	7	1.123	5	1.084	4
	*18S*	1.338	4	1.133	5	1.011	4	0.633	1
	*28S*	1.250	3	1.165	6	0.837	3	0.701	2
	*GAPDH*	1.740	7	0.977	4	1.498	7	1.374	7
	*EF1α*	1.494	6	0.869	1	1.132	6	1.228	6
	*TBP*	1.145	1	0.876	2	0.622	2	0.633	1
	*RPL7*	1.176	2	0.912	3	0.563	1	0.830	3
Pooled samples	*ACT*	2.941	6	2.033	6	2.611	6	1.585	6
	*18S*	1.946	2	1.316	3	1.313	2	0.864	3
	*28S*	2.382	4	1.910	5	1.985	4	1.126	4
	*GAPDH*	1.952	3	1.150	2	1.356	3	0.661	2
	*EF1α*	3.523	7	1.729	4	3,286	7	1.883	7
	*TBP*	1.689	1	1.129	1	0.889	1	0.389	1
	*RPL7*	2.543	5	2.115	7	2.194	5	1.329	5

For diferent developmental stages, the stability rankings were almost the same using ΔCt method and NormFinder with *TBP* and *EF1α* as the top two most stable candidate reference genes. GeNorm analysis identified *28S* and *EF1α* as the most and the least stable reference genes, respectively. *GAPDH* and *EF1α* were demonstrated as the top two most stable reference genes by Bestkeeper (Table 2). Integrating the results from all four programs, RefFinder provided a ranking as *28S*, *RPL7*, *TBP*, *18S*, *EF1α*, *GAPDH* and *ACT* (Fig 2B) across different developmental stages. In comparison with the cut-off value of 0.15, the pair-wise value of V_6_/_7_ was above 0.15 (Fig 3), suggesting the number of reference genes should be added for accurate normalization.

For three different regions, the top two most stable reference genes were identified as *TBP* and *RPL7* by the ΔCt method and NormFinder, respectively. Analysis using BestKeeper showed that the most stable reference gene for normalization was *EF1α*. According to RefFinder, the ranking of seven selected reference genes from the highest to lowest stability) was: *TBP*, *18S*, *28S*, *RPL7*, *ACT*, *EF1α*, and *GAPDH*. GeNorm showed that all the pair-wise values of V_n/n+1_ were below 0.15 across different regions including Xinxiang, Kaifeng, and Luohe (Fig 3).

For different temperatures, the ΔCt method and NormFinder results exhibited that *18S* and *ACT* were considered as the most stable and the least reference gene, respectively. GeNorm results revealed that *28S* and *GAPDH* were the top two most stable reference genes. The expression stability of the seven reference genes from the most to the least stable was comprehensively ranked as *28S*, *GAPDH*, *TBP*, *18S*, *RPL7*, *EF1α* and *ACT* based on RefFinder (Fig 2C). From geNorm analysis, the V_n/n+1_ values were all below 0.15 across different temperatures (Fig 3), indicating no supplemental reference gene was added for normalization of the qRT-PCR analyses. Thus, *28S* and *GAPDH* were the most suitable candidate reference genes across different temperatures.

For three different regions, the top two most stable reference genes were identified as *TBP* and *RPL7* by the ΔCt method and NormFinder, respectively. Analysis using BestKeeper showed that the most stable reference gene for normalization was *EF1α*. According to RefFinder, the ranking of seven selected reference genes from the highest to lowest stability) was: *TBP*, *18S*, *28S*, *RPL7*, *ACT*, *EF1α*, and *GAPDH* (Fig 2D). GeNorm showed that all the pair-wise values of V_n/n+1_ were below 0.15 across different regions including Xinxiang, Kaifeng, and Luohe (Fig 3).

For herbicide application, the most stable reference gene was *TBP* based on ΔCt method and geNorm. *TBP* and *28S* were the top two ranked genes by geNorm (Table 2). However, *EF1α* was the least stably expressed reference gene by employing all the four different algorithms. Based on RefFinder, the order of reference gene stability ranking across herbicide application was: *28S*, *TBP*, *RPL7*, *GAPDH*, *18S*, *ACT* and *EF1α* (Fig 2E). GeNorm showed that the V_n/n+1_ values were all below 0.15 across herbicide application (Fig 3), indicating supplemental reference gene was unnecessary. Therefore, *28S* and *TBP* were considered the top two suitable reference genes across herbicide stress.

For all the experimental conditions, the ranking order of reference gene stability was the same according to Ct method and NormFinder, and *TBP* and *EF1α* were confirmed as the most stable and the least stable one, respectively. Furthermore, *ACT* was confirmed as the less unstable reference gene by all of them (Table 2). Based on RefFinder, the stability ranking order of reference gene across various conditions was: *TBP*, *GADPH*, *18S*, *28S*, *RPL7*, *ACT*, and *EF1α* (Fig 2F). All pairwise variations with the V_n/n+1_ values were evaluated using GeNorm across diverse conditions (Fig 3). Therefore, *28S* and *RPL7* were determined to be the best housekeeping genes under various conditions.

qRT-PCR has been broadly used for quantification of the gene transcript abundance. The accurate and reliable qRT-PCR results were always affected by diverse factors including RNA quality, cDNA synthesis, experimental procedures, and reference gene normalization. Therefore, reliable reference gene selection is of particular importance for successful qRT-PCR analysis. To date, no study have systematically been focused on evaluation of the reference gene stability in BGA.

Our results indicated that *28S* and *RPL7* were the top two most stable reference genes across various experimental conditions according to RefFinder analysis and four diferent algorithms including ΔCt method, geNorm, BestKeeper, and Normfinder. The ranking order of reference gene stability can vary due to different algorithms with the four analytical tools. With the integration the four statistical methods, RefFinder, a comprehensive web-based tool, was chosen to rank the overall stability of the seven candidate reference genes. In this study, *28S* and *RPL7* were considered as the top two most stable reference genes using RefFinder within specific condition. However, *GAPDH*, *ACT* and *EF1α* were identified as the unstable reference genes across development stages, tissues, temperatures, regions, and herbicide treatments, sexes. Similarly, *18S r RNA* or *28S* was identified as the most stable reference gene in rice[24], the Australian sheep blowfly[25], and eggplant[26]. In contrast, *GAPDH* was reported as one of the most stably expressed genes in weed species *Lolium* sp.[27] and *Avena fatua*[28]. *GAPDH* was also identified unstable in *Petunia hybrida* across development stage[29]. *EF1α* was considered to be the reliable reference gene under given experimental conditions in current study, and this is the same as results of some insects such as bumblebee[30] and cotton bollworm[31].

Liu et al.[28] evaluated TBP as the best reference gene in *Avena fatua*, and TBP was also identified as an ideal reference gene in tomato [32] and annual ryegrass[33]. Our results demonstrated that traditional reference gene *ACT*, involved in cytoskeleton structure, was not usually unchange under diverse experimental treatments. Similar findings were verified in the beetle *Tribolium castaneum*[34], perigord black truffle[35] and *Avena fatua*[36]. Therefore, no reference genes in multiple species show unchanged expression across all the tested conditions[37,38], suggesting that it is of particular important to identify suitable reference genes in BGA.

### Validation of selected reference genes

**Fig 4:**
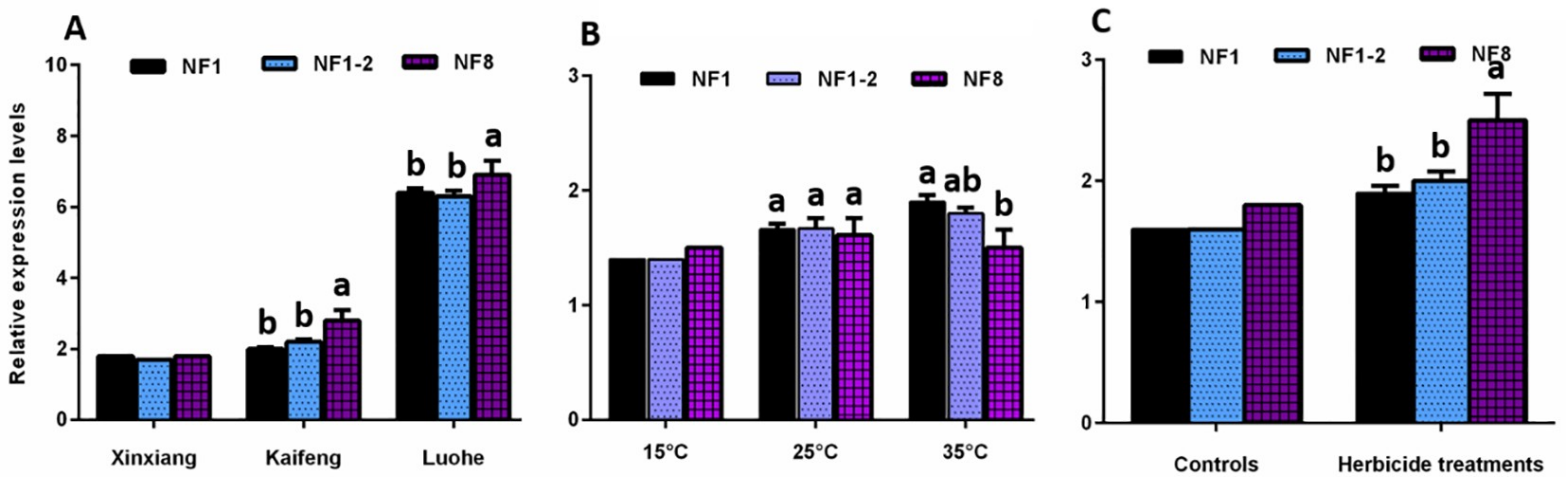
Expression patterns of *HSP70* using different normalization factors. (A) in different regions. (B) in different temperatures. (C) under herbicide treatments. Bars represent the mean ± standard deviation of 3 biological replicates.

To validate the recommended reference genes in this study, the relative expression level of one target gene, *HSP70*, was analyzed under all the experimental conditions. For different regions of BGA, the relative expression level of *HSP70* was significantly up-regulated in Luohe and Kaifeng than in Xinxiang when using the most stable reference gene (NF1: *TBP*), the recommended normalization combination (NF1–2:*TBP* and *18S*), and the least stable reference gene (NF7: *GAPDH*), significant difference was found in *HSP70* expression when using single reference gene (the most stable, *TBP*) or reference gene combination (NF1–2: *TBP* and *18S*) and single reference gene (the least stable, *GAPDH*) (Fig 4A).

For BGA exposed to different temperatures, the relative expression level of *HSP70* expression was significantly higher at 25°C or 35°C than at 15°C. The relative expression levels of *HSP70* increased less at 35°C using the least stable reference gene (*ACT*) in comparison with the most stable one (*28S*) and the recommended normalization factors (*GAPDH* and *28S*) as normalization (Fig 4B). Normalized by stable reference gene, the combination of the two most stable reference genes or the least stable reference gene, significant difference was found in *HSP70* expression when treated with herbicide when using single reference gene (the most *28S*) or reference gene combination (*28S* and *TBP*) and and single reference gene (the least *EF1α*). Meanwhile, *HSP70* was significantly up-regulated under the herbicide stress in comparison with the control groups. The expression level of *HSP70* was significantly increased by normalization with the least stable reference gene (*28S*) than by the most stable reference gene (*GAPDH*) and the recommended combination (*ACT* and *GAPDH*) (Fig 4C).

*HSP70*, an important stress-inducible heat shock protein gene, is as a target gene to validate its relative expression using the most stably expressed reference gene, the most unstably expressed one, and the recommended combination. The qRT-PCR analyzed results indicated that the transcriptional levels of target gene *HSP70* varied significantly with normalization of different reference gene. This study represents the first attempt to select a set of candidate reference genes in BGA under various conditions for the normalization of gene expression data using qRT-PCR. *28S* and *RPL7* were identified as the most stably expressed reference genes under all experimental conditions, which provided a standardized procedure for the quantifcation of gene expression in BGA.

## Supporting information

S1 File Sequences of reference and target genes tested in *Galium aparine*. (XLS)
